# The NICE Medical Technologies Evaluation Programme (MTEP): manufacturer submission challenges

**DOI:** 10.1258/jrsm.2012.120042

**Published:** 2012-04

**Authors:** Kirsty Sprange, Maxine Clift

**Affiliations:** Healthcare Innovation and Technology Evaluation Centre (HITEC), Derby Hospitals NHS Foundation Trust London Road Community Hospital, London Road Derby DE1 2QY, UK

## Introduction

The purpose of this report was to explore and describe the challenges that healthcare sector manufacturers face when producing a submission to the NICE Evaluation Pathway (EP) Programme for medical technologies. The EP Programme was developed to evaluate new and innovative medical technologies to inform adoption of efficient and cost effective devices and diagnostics in the NHS. The premise was to produce recommendations based on a similar process currently used for pharmaceutical products. A submission requires manufacturers to complete a data capture template collating high quality clinical and cost effectiveness evidence for their device. This includes indicating how the device integrates into current NHS clinical pathways and how it performs in relation to current best practice. The time scale for completion of a submission is an approximated 6 weeks. Particular consideration was given to small and medium enterprises (SMEs), and availability and access to resources and skills.

A consultation process was undertaken to ascertain the opinion of small, medium and large manufacturers in the healthcare sector who could potentially submit an application to the EP programme. The results of this process would highlight any real or perceived barriers identified by manufacturers that could prevent them from submitting to the new EP programme.

The EP programme was renamed after preparation of this report to the Medical Technologies Evaluation Programme (MTEP), and details are available here: http://www.nice.org.uk/mt.

## Methods

### Focus groups

Focus groups were employed to ascertain opinion from SME's, large manufacturers, start-out companies and intermediaries. In this instance an intermediary was defined as an organization that provides support functions to manufacturers including regulatory approval, research and marketing.

A training day was developed and organized by the Healthcare Innovation and Technology Evaluation Centre (HITEC) and hosted by Medilink East Midlands, a specialist support organization for the healthcare sector. The day was attended by members of their organization which is represented by the following areas of the healthcare community; medical technology, biotechnology, pharmaceuticals, Universities and the NHS.

The morning session consisted of presentations from both NICE and HITEC outlining the submission process to manufacturers. It was assumed the presentations would help inform the discussion for the workshops in the afternoon session. The afternoon session consisted of three interactive workshops using the focus group technique to encourage discussion within homogenous manufacturer groups. The selected methodology reduced the issue of bias within groups, for example one manufacturer dominating the conversation, and ensured results could be generalized to the size of manufacturer.

The four groups were constructed as follows:

- Group 1 – SMEs (*n* = 6);
- Group 2 – Large manufacturers (*n* = 5);
- Group 3 – Start-out companies (*n* = 5);
- Group 4 – Intermediaries (*n* = 5).

The workshops were developed to explore what challenges manufacturers felt their organization may face in relation to the following three topics:

1. Information accessing skills;
2. Evaluating, interpreting and presenting the evidence;
3. Access to resources.

Each workshop was set a specific task in the form of a question with four discussion points designed to encourage dialogue and debate within each homogenous group. Each group remained the same throughout and took part in all three workshop sessions to maximize resulting data. This method ensured opinion could be attributable to a particular manufacturer size for the purposes of analysis.

The individual groups were asked to record their discussion as bullet points on a flipchart which were then collected at the end of the afternoon session. These were then typed-up verbatim and thematic analysis used to identify key themes. The individual groups were then asked to feedback to the whole group and discussion was facilitated by the researchers. Results of the general discussion were summarized and written up on a flipchart during the discussion by one of the researchers. This was undertaken in front of the groups and with their consent to ensure reliability. Thematic analysis was also undertaken on these data and included in the overall analysis.

Focus groups accessed qualitative in-depth opinion from manufacturers including SMEs, large manufacturers, spin out companies and intermediaries. The methodology allowed open discussion about the skills and knowledge requirements for the submission process within homogenous groups. This enabled conclusions to be more generalised and reduced potential bias.

### Questionnaire survey

A questionnaire was developed and conducted with members of Medilink East Midlands and a cross-section of device manufacturers who had worked with HITEC on previous projects. Space restricts reproduction of the 26 questions here. A copy of the questionnaire is available by contacting NICE (email: medtech@nice.org.uk with ‘RSGT222′ in the subject line). The questionnaire was divided into 4 sections designed to explore different aspects of the submission content and process. These were:

- Information accessing skills;
- Evaluating and interpreting the evidence;
- Organizational access to resources.

To enable data analysis to be conducted using homogenous groups, manufacturers were asked to indicate their company size (small, medium or large) based on the definition set out by the European Commission.^1^ Manufacturers completing the questionnaire survey had no prior experience of the EP programme submission template, therefore the questionnaire survey was specifically designed to account for this lack of knowledge.

The questionnaire survey was conducted with a number of manufacturers of varying sizes and were UK companies or had representation in the UK. Manufacturers were identified and contacted in two ways, either by post or by email, and could return the completed questionnaire survey by free post or email for convenience. Manufacturers were identified from the Evaluation Assessment Centre's (EAC) own contact database and also through the membership of Medilink East Midlands. A total of 9 completed questionnaires were returned for analysis and 1 manufacturer provided comment, but did not complete the questionnaire.

Manufacturers were provided with an invitation letter explaining the purpose of the questionnaire survey and why they had been contacted. A brief description of the EP programme was provided in the letter including a link to the new EP programme website for further information. Also included was a summary of the NICE EP programme submission template specifically designed and produced by the Centre as an aide to completion of the questionnaire. Manufacturers were given a period of 3 weeks in which to complete and return their questionnaire survey and a reminder was issued 5 days prior to the final deadline. SPSS (v17.0) was used to collate and analyse the results of the questionnaire survey.

## Results

### Focus groups

Identifying and accessing appropriate academic papers was considered a potential barrier to submission. For the SME group in particular, the whole process of identifying articles through searchable databases, ordering and obtaining articles then evaluating and presenting evidence was considered costly and time consuming in relation to the benefit of a submission. This concern was reiterated by the start-out company group. The intermediary group also reaffirmed that based on their own experience of working with SMEs, they would find the process of accessing academic papers difficult in terms of cost and access.

Although the large manufacturers identified several relevant searchable databases including Medline and Cochrane, their actual knowledge of the databases was limited or flawed. They considered both databases to have a pharmaceutical focus, with either no or limited medical device content. The same group also indicated they felt journals very often did not include articles on new and innovative technologies, adding to the burden of trying to find high quality supporting evidence.

Overall it was agreed that much of the terminology encountered such as search strategy, PICO, CASP tools and WINBUGS was new to the groups represented. They had no prior knowledge of these terms, their meaning or in what context they would be used. It was also noted that collating data of this type, that of clinical and cost effectiveness evidence, was not an activity they undertook regularly as it was not a requirement for other aspects of device development, for example obtaining regulatory approval and marketing.

The general lack of available supporting data was also raised by the SME group. They stated that new and innovative devices by their very nature would not have large amounts of supporting data because of the fact that they are new and innovative. They suggested conversely that if a device had a lot of supporting data, such as clinical trials, then it was no longer considered new and innovative to the market. General discussion suggested that studies and trials if conducted at all, were carried out as part of the post-marketing period of a device. SMEs also noted, and it was reiterated by the larger manufacturer group, that when a device is new and innovative it most likely has no comparator for the purposes of the submission. The submission process was therefore considered imbalanced in relation to its development for new and innovative technologies in comparison to its request for high quality evidence.

The focus of UK trial data over other international trial data was also discussed. This issue was raised specifically in response to information provided by the NICE presentations during the morning session. The comment was made by the SME group who felt that all evidence, regardless of what country the trial was conducted in, should be considered relevant for a submission.

Cost was raised as a particular issue for the SME group who implied that the cost in human resources to undertake a submission would be a key factor in their decision to submit or not. Costs were identified as related to either the allocation of in-house staff or the buying-in of consultancy which would significantly increase costs further. This issue was re-iterated by the start-out companies who stated that inexperience would cost time and money in terms of learning how to identify, access and evaluate the evidence for a submission, and also accessing the specialist skills required, for example to produce an economic model. The intermediary group added that in their experience, these costs may not be seen as an efficient use of resources, especially for SMEs.

SMEs also stated that smaller companies would only conduct small trials or evaluations rather than higher quality RCTs due to the increased costs. The group as a whole noted that accessing funding such as grants to support research activities or undertake their own trials was complex and limited. In light of this, distribution of available funding had to be directed on a need basis. In this case, manufacturers stated they would direct available funds into developing, producing and marketing their device. Priority would not be given to conducting research trials. This relates back to the issue of the types of available evidence to support a submission for a technology, particularly one developed by a smaller manufacturer with access to fewer resources.

The timescale for completion of a submission was considered inflexible and could potentially prevent manufacturers from submitting an application if they considered it not within their abilities. It was agreed that the ability to work to the 6-week timescale would be dependent on the available resources, skills and knowledge of each individual manufacturer.

The group also queried how and whether the approach selected by NICE, that of the EP programme submission, was relevant to medical devices. Unlike pharmaceuticals which remain as market leaders for extensive periods of time, manufacturers indicated that the medical device market changed rapidly and devices were regularly superseded by new devices within short periods of time.

Both the SME and intermediary groups raised the issue of affordability, particularly for smaller manufacturers. They questioned whether there was any cost benefit in either trying to undertake a submission themselves without the necessary knowledge and skills, or to outsource the submission at a significant cost. Manufacturers declared they did not fully understand the purpose of the new pathway, the reasons it had been established and why they should submit their device. This was particularly highlighted in relation to the new coalition government and the future role of NICE.

In particular SMEs indicated that, if there was no direct connection to NHS purchasing by obtaining NICE approval through this pathway, they could not perceive any benefit from submitting. Manufacturers also stated they currently accessed NHS purchasing and clinicians to market their products already, therefore the purpose of the EP programme was unclear, particularly in relation to the commitment of already limited resources. The manufacturers asked for further clarity regarding the benefit of making a submission. They stated that, without understanding the value of obtaining NICE approval in real terms, it would be very difficult to justify allocating funds to produce a submission.

A need was also identified to bridge the gap between encouraging innovation and administration in terms of costs to the manufacturer. The submission process itself was seen as a barrier due to the large amount of paperwork required to support a submission. It was seen as ‘extra work’ in addition to the existing work undertaken for regulation and marketing. The groups indicated that although innovation was encouraged, the bureaucracy they encountered was actually a hindrance rather than a help.

All groups stated risk was a major concerning factor to submission to the EP programme. In particular small manufacturers stated they would need to weigh up the risk of submitting their device and potentially receiving a negative evaluation. Not only would the submission have incurred the manufacturer costs in time and money, but also in relation to their reputation and that of their device and their future ability to access funding or supply the NHS.

### Questionnaire survey results

Out of the 9 manufacturers that responded:

– Small manufacturers (*n* = 6);
– Medium manufacturers (*n* = 1);
– Large manufacturers (*n* = 2).

### Section 1 – Information accessing skills

All respondents stated that their company accessed and read published academic journals, 44% weekly, 33% monthly and 22% as and when required. This access was for a wide range of reasons covering their own medical device(s), comparative device(s) and for literature searches. One respondent also stated they accessed academic journals for ‘continuing education in our professional fields, awareness of state of the art [technologies].’

Regarding access to academic journals, 100% of respondents stated they sourced and used academic journals. Of these, 78% of respondents subscribed via organizational subscriptions directly to journals, 56% used libraries and public access journals and 11% used a personal subscription. All respondents used either company subscriptions or public access journals.

In relation to undertaking literature searches and using search strategies and techniques, 78% of respondents did state that they had conducted literature searches. Furthermore, 67% stated they had never developed a search strategy. However, no responders had used a recognized methodology such as PICO. The companies that had not carried out a literature search stated this was either due to not having needed to or due to lack of access to appropriate searchable databases. The reason ‘don't know how to complete a literature search’ was not selected, this suggests companies could carry out literature searches when required.

There was mixed evidence for use and knowledge of searchable databases. Medline, the Cochrane Library, Pubmed and the NHS National Library for Health were the most accessed, with the majority of respondents having heard of and used them. There was some knowledge and use of Embase, Cinahl and OVID. Few of the responders stated they had heard of EconLit, NHS EED or PsychInfo. Companies indicated they used these databases daily and weekly (33%) and monthly (44%) with other responders using databases as required. All responders used the databases for work purposes. The databases were accessed by different means; 44% via free subscriptions, 44% using organization subscriptions and 44% accessing the National Library for Health (NHS) website. Only one company used a personal subscription.

Filtering mechanisms had been applied by 67% of responders. Of the 33% who had not used filters, half stated that although they were aware of them they did not use them due to a lack of understanding and knowledge on how to apply them. The respondents who had applied filtering mechanisms did have knowledge of one or more of the following indexing and cataloguing systems: MeSH Headings, Emtree, Boolean Operators and Wildcards. The companies that stated they had not used filtering mechanisms were unaware of any of these systems.

### Section 2 – Evaluating and interpreting the evidence

Only 33% of the companies had used an appraisal tool to evaluate evidence including *n* = 1 Critical Appraisal Skills Programme (CASP) and *n* = 2 Centre for Evidence Based Medicine (CEBM). None of the companies had used any reporting tools to summarize literature or article evidence, although 33% of companies stated they had undertaken evidence synthesis. The companies who had not carried out evidence synthesis stated this was due to a lack of knowledge and necessary skills.

Respondents were asked to state the level of knowledge they had of clinical research design methodologies. Of the respondents, 56% indicated they had a working knowledge of a number of the methodologies included in the questionnaire survey such as randomized and non-randomized controlled trials, clinical trials, comparative trials and case studies. The remaining 44% had only a limited knowledge or no knowledge of the listed methodologies. Less than 33% of respondents stated they had a working knowledge of systematic reviews, meta-analysis or economic modelling.

A total of 56% of respondents had developed an economic model which used a cost benefit or cost-consequence model and Microsoft Excel. One large manufacturer also indicated they had developed a budget impact model. No use of specialist or statistical software was reported. Only 33% of the companies had any working knowledge of the following evaluation and economic model terms: budget impact analysis, progression-free and post progression-free survival, incremental cost-effectiveness ratio (ICER), time horizon, transition probabilities, health state, cycle length, half-cycle correction and probabilistic/deterministic sensitivity analysis. Of manufacturers surveyed, 50% indicated a working knowledge of sample sizes, hypotheses and variables. However, there was limited working knowledge (less than 50% of companies) of descriptive statistics and statistical techniques such as Power Calculations, Analysis of Variance (ANOVA), Chi-squared, Mann-Whitney, t-tests, Spearman's rank correlation coefficient and regression analysis.

### Section 3 – Organizational access to resources

Companies were asked if they had specific budgets to carry out certain tasks. Respondents were able to give multiple responses. Only 33% of the respondents had specific budgets to develop and conduct literature searches, carry out critical appraisals of the literature and write written reports. Only two manufacturers had a designated budget to develop economic models and these were both the large manufacturers represented in the questionnaire survey. However, 63% of companies had no identified or ‘ring fenced’ budget for any of these activities.

A wide range of primary research activities covering RCTs, clinical trials, cohort and comparative studies, clinical and cost effectiveness studies and systematic reviews were carried out by 56% of respondents either in-house or externally. The other 44% did not carry out any of the listed primary research activities. The results showed that 78% of the companies did indicate they would have staff available, where necessary, to produce a submission to the NICE EP Programme for Medical Technologies. However, only 22% of the companies believed it was possible to complete the EP programme submission within the six-week timeframe. Reasons provided included ‘gaining economic evidence taking much longer’ and ‘lack of full understanding of the process’.

All of the companies stated they had access to clinical specialists in the medical field in which their device would be used. A further 89% stated they had access to NHS specialists, 67% had in-house clinical specialists and 56% had access to clinical specialists within private healthcare. Access to research specialists was mixed as 22% of the companies reported no access to any specialists, and the other 78% had a mixture of in-house and external access to researchers, clinical librarians or information scientists and staff with research qualifications. Only one large manufacturer had access to an in-house health economist. None of the manufacturers had in-house medical statisticians although 67% had external access to medical statisticians and 44% to health economists.

### Section 4 – Medical devices in context

There was a mixed response to whether manufacturers were able to describe how their devices fit in with the current NHS clinical pathway of care. Indicatively, 78% of companies stated their device could be incorporated into the existing clinical care pathway and of these, 57% stated their device would change the existing pathway. Requirement for other therapies to supplement their device in the pathway was indicated by 33% of respondents, and 33% stated that additional tests would be required. Only one large manufacturer stated that other facilities, infrastructure or additional administrative support would be required compared with the existing clinical pathway.

The NICE submission pathway requires companies to clearly describe the whole life costs related to their medical device(s). All of the manufacturers stated they had information regarding unit costs and selling price, and where applicable consumable costs and lifespan. However, where applicable, 40% of respondents stated they did not have information regarding average costs, average frequency of use, average length of use per treatment and service or maintenance costs.

The outcome of the focus groups and questionnaire survey was a set of recommendations to enable NICE to encourage and support manufacturers, in particular SMEs, with completion of applications to the EP programme. Although the list of recommendations has not been included in this report, they are referred to within the conclusion.

### Conclusions

Due to the multinational market for medical devices and the significant presence and contribution of SMEs within this innovation landscape, it is imperative that NICE engage constructively with SMEs to ensure their active involvement in the new EP programme. The first barrier, however, is the current economic climate; with reduced investment in the medical technology sector and increasingly limited budgets, allocation of resources to innovation activities will inevitably suffer. NICE need to acknowledge and address, where possible, the actual and perceived barriers observed by manufacturers to ensure manufacturers see submission to the EP programme as a viable and beneficial exercise.

Engaging non-innovator companies will be the greater challenge for NICE, compared to companies already proactive in undertaking research and innovation activities. Improved support functions and guidance should alleviate some of the administrative barriers. The major issue which could potentially prevent a submission, that of understanding the purpose of the EP programme, appears to be consistent across all manufacturers. There is a real need for improved marketing. In particular, accessible information on why the programme exists and how manufacturers will benefit from a submission in real terms. This may appear an easy barrier to overcome through the conduct of a structured marketing strategy, however in the current economic climate, manufacturers consulted as part of this review stated there would need to be a significant cost-benefit if they were to commit already limited resources to producing a submission. This was the same for both internal resources and consultancy. The EP programme was seen as a significant risk by manufacturers, particularly by SMEs if they obtained a negative report from NICE, which would be costly not only in terms of resources committed, but also company and device reputation. NICE need to have a clear and focused purpose and strategy for the EP programme which can be delivered in a suitable format to all manufacturers. Building strong relationships with professional bodies and healthcare sector organizations such as the Medilink network, would enable dissemination of the programme to large groups of manufacturers through their membership.

Significant barriers to the EP programme can be drawn in parallel with those affecting innovation activities in general. The EP programme is itself an innovation activity for manufacturers in relation to device evaluation and research. Previously identified barriers have included cost, knowledge, the marketplace and regulation. Cost and knowledge are particularly relevant to the EP programme and were clearly identified as potential barriers within the consultation process. Access to funding to support research activities was considered complex and time consuming with only limited chances of success. Costs were also identified in staff time, access to databases and articles, and consultancy fees. The consensus opinion of the focus groups was that the EP programme was seen as a further cost to the manufacturer, in addition to regulatory applications, device production and marketing. In particular, several SMEs indicated they would not consider a submission to the EP programme due to these related costs. Although NICE cannot provide direct funding to manufacturers to assist with a submission to the EP programme, they can provide improved support functions and tools to enable manufacturers to undertake the review of evidence themselves. By providing information, guidance and access to resources such as critical appraisal tools and searchable databases, NICE can empower medical device manufacturers to obtain the skills and knowledge required to complete a submission. As part of the recommendations resulting from this review, NICE may also consider providing a series of dissemination activities such as awareness or training sessions to inform manufacturers about the process and the evidence requirements. Both of these recommendations could potentially alleviate some of the need for external consultancy costs and save the manufacturer time in trying to identify and access these resources with no prior knowledge or experience.

Manufacturers represented at the focus groups also raised concerns regarding the appropriateness of the EP programme for use with medical devices. In particular they suggested there was too much comparison with the pharmaceutical industry on which assumptions had been made regarding available evidence and ability of medical device manufacturers to undertake clinical trials. The two markets, pharmaceutical and medical technologies, were also considered very different by manufacturers. They indicated that significantly less investment was made in medical technologies, and compared to pharmaceuticals it was a rapidly changing innovation landscape with new devices continually entering the market. NICE need to keep abreast of the medical device innovation landscape and ensure the EP programme is fit for purpose; that of evaluating new and innovative medical technologies. The lack of awareness of the programme prior to the focus groups may have had an affect on manufacturer responses. Although manufacturers received presentations detailing the EP programme during the morning session of the training day, they still felt the process was unclear when undertaking the workshop sessions in the afternoon. This demonstrates the need for improved communication between NICE and the manufacturing community regarding the purpose of the EP programme, and why and how it was developed. The design and implementation of a clearly defined marketing strategy has been included in the report recommendations.

The consultation process as a whole, raised a number of significant issues in relation to basic and general research skills that need to be addressed by NICE if they are to encourage submissions from manufacturers, in particular SMEs. The spectrum of research skills required for a submission were distinctly lacking within the manufacturers represented. Knowledge and skills gaps were identified for developing search strategies, knowledge of and accessing searchable databases, accessing other resources including funding and specialists, and awareness and ability to use appropriate tools including critical appraisal tools and IT packages. Manufacturers indicated they would not be able to fulfil the requirements of a submission because they did not possess the fundamental skills necessary. As a potential barrier, this issue poses several problems. Although improved information resources and support functions could be provided, such as a web-based tool kit for manufacturers detailing where and how to access resources and training, it is not possible for other factors such as funding to be made available through NICE. The toolkit could include details of potential funding streams to assist manufacturers.

A balance is required in the EP programme. The medical technology landscape has been seen to undergo rapid change meaning rapid assessment and evaluation is required to stay current. This needs to be balanced however, with the identified lack of research skills within the medical technology sector and how this will affect ability to not only complete a submission but also whether a six-week timescale is achievable. Of the manufacturers surveyed, less than a quarter believed it possible to complete a submission to the EP programme within the given timeframe. This review has identified and described a number of issues and barriers that could have a direct effect on submissions to the EP programme. In response, a number of recommendations have been developed to address these barriers where possible. All of the recommendations made have been designed to alleviate a real and perceived burden from the manufacturer, and to encourage their engagement in the EP programme ultimately resulting in increased submissions.

## DECLARATIONS

### Competing interests

The authors have no competing interests to declare. All articles were originally commissioned by NICE from independent External Assessment Centres (EACs) to address areas of interest to the Medical Technologies Evaluation Programme (MTEP, originally known as Evaluation Pathways EP). The EACs were engaged under a contract to NICE

### Funding

NICE

### Ethical approval

Not applicable

### Guarantor

Chris Pomfrett

### Contributorship

Kirsty Sprange and Maxine Clift wrote the first version of the manuscript while working for HITEC, an independent academic External Assessment Centre working under contract to NICE.

Dr Chris Pomfrett does not claim authorship, but acted as supervising editor for this work in NICE. Dr Chris Pomfrett is a Technical Adviser working in the Medical Technologies Evaluation Programme (MTEP) at NICE

### Acknowledgments

None
